# Prediction of DNA-Binding Protein–Drug-Binding Sites Using Residue Interaction Networks and Sequence Feature

**DOI:** 10.3389/fbioe.2022.822392

**Published:** 2022-04-20

**Authors:** Wei Wang, Yu Zhang, Dong Liu, HongJun Zhang, XianFang Wang, Yun Zhou

**Affiliations:** ^1^ College of Computer and Information Engineering, Henan Normal University, Xinxiang, China; ^2^ Key Laboratory of Artificial Intelligence and Personalized Learning in Education of Henan Province, College of Computer and Information Engineering, Henan Normal University, Xinxiang, China; ^3^ Computer Science and Technology, Anyang University, Anyang, China; ^4^ Computer Science and Technology, Henan Institute of Technology, Xinxiang, China

**Keywords:** residue interaction network, extreme gradient boosting, binding site, sequence, protein–ligand

## Abstract

Identification of protein–ligand binding sites plays a critical role in drug discovery. However, there is still a lack of targeted drug prediction for DNA-binding proteins. This study aims at the binding sites of DNA-binding proteins and drugs, by mining the residue interaction network features, which can describe the local and global structure of amino acids, combined with sequence feature. The predictor of DNA-binding protein–drug-binding sites is built by employing the Extreme Gradient Boosting (XGBoost) model with random under-sampling. We found that the residue interaction network features can better characterize DNA-binding proteins, and the binding sites with high betweenness value and high closeness value are more likely to interact with drugs. The model shows that the residue interaction network features can be used as an important quantitative indicator of drug-binding sites, and this method achieves high predictive performance for the binding sites of DNA-binding protein–drug. This study will help in drug discovery research for DNA-binding proteins.

## 1 Introduction

DNA-binding protein plays a crucial role in many biological processes, such as regulating gene expression, DNA duplication, DNA recombination, DNA repair, histone modification, and other biological activities associated with DNA (Ptashne, 2005; Audia and Campbell, 2016; Luscombe et al., 2000). Identifying these proteins is beneficial to find out the cause of disease for most medical researchers, which helps them pinpoint the cause of the disease. BRD4 is a DNA-binding protein that has attracted wide attention in the field of anticancer drugs. The suppression of BRD4 is not only an effective way to cut off the communication between super-enhancers and target promoters but also represses the expression of oncogenes subsequently, which is related to cancer cell death (Lu et al., 2020). DNA-binding protein 43 is the culprit for amyotrophic lateral sclerosis (ALS). The unusual accumulation of DNA-binding protein 43 in motor neuron cells leads to neurotoxicity, which is a pathological hallmark of several other neurodegenerative diseases (Watanabe et al., 2020). Another research has found DNA-binding protein A (dbpA) may be a new and effective therapeutic target, which is useful for colorectal cancer (CRC). The downregulation of dbpA is a pivotal method to inhibit cell proliferation and induce cell apoptosis as well as cell cycle arrest in cancer cells because it can not only restrict the growth of tumor but also improve the drug sensitivity of CRC cells *in vivo* (Tong et al., 2020). These studies have shown that DNA-binding proteins exist in living cells widely and participate in many cell activities. Then, the predictive studies of DNA-binding proteins are key tasks in drug development and treatment of diseases for most researchers (Rahman et al., 2018; Wang et al., 2021).

With the continuous development of biotechnology, it has become important to understand protein functions and drug discovery to predict the protein–ligand binding sites. In the past years, structure-based, sequence-based, and hybrid system method (both sequence and structure characteristics), etc. were used to predict protein–ligand binding sites, among which the ligand binding sites of the established 3-demensional protein structure can be effectively forecasted by structure-based methods (Xie and Hwang, 2015; Allen et al., 2015; Wu et al., 2018). The molecular docking has also been widely regarded for its function in finding ligand binding sites (Wu et al., 2018). Considering the protein structures are few in number to satisfy the growing demand, sequence-based methods were applied in predicting the protein–ligand binding sites directly (Yang et al., 2013; Ding et al., 2017; Zhao et al., 2019). For instance, Ding (Ding et al., 2017) obtained the position-specific scoring matrix feature through the protein sequence, and subjected the feature to discrete cosine transform, and then obtained the PSSM–DCT feature, and finally used under-sampling and ensemble classifier to build a prediction model. In addition, there are some methods that combine sequence and structure information to obtain better performance of prediction (Liu and Hu, 2011; Lu et al., 2019). For example, HemeNet (Liu and Hu, 2011) has demonstrated that hybrid models working together will achieve a better performance in specific prediction of HEME binding residues than that of single prediction.

It is known that structure method and structure/sequence method can predict the results accurately than sequence method. But, due to the lack of three-dimensional structures, this structure/sequence-based method and structure-based method is limited. Herein, we attempt to invent an innovative computational method which is based on DNA-binding protein sequences and network topological characteristics to identify drug-binding sites. Also, although these advancements have been made in protein–ligand binding sites predictions, the research level in predicting DNA-binding protein–drug ligand binding sites is still at the initial stage. At the same time, the research studies on the DNA-binding protein–drug-binding sites based on bioinformatics method are very few at present. We manually screened 120 DNA-binding protein–drug complexes to construct the data set for this study. Also, we look forward to clarifying the intrinsic correlation between DNA-binding protein and drug interactions through identifying the drug-binding sites of DNA-binding proteins.

**FIGURE 1:**
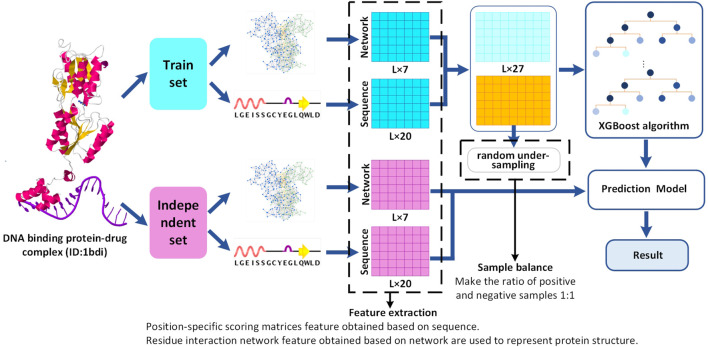
Flowchart of DNA-binding protein–drug-binding sites prediction. Red represents the independent set data, blue represents the training set, light blue, and yellow represent the positive and negative sample data of the training set, respectively. A reference data set of 120 DNA-binding protein–drug complexes is generated from sc-PDB. The characteristic set is composed of network and sequence feature, and a prediction model is constructed by XGBoost. Finally, the performance of the training data set and the independent data set is evaluated.

In this study, we consider three popular classifiers (XGBoost, SVM, and CART) for conducting research on the prediction of drug-binding sites using the DNA-binding protein–drug complexes. To search the most suitable predictor, three different machine learning methods (XGBoost, SVM, and CART) are used to predict the binding sites for drugs comparatively by utilizing the DNA-binding protein–drug complexes, and the best one is chosen by us. Through comparative research, the 20-dimensional position-specific scoring matrix feature and the 7-dimensional residual interaction network feature as a preferable feature set are selected to improve the proposed predictor. The XGBoost-based method proposed in this study shows better AUC and ACC scores on either the training data set or the independent data set. The working flowchart of the proposed DNA-binding protein–drug-binding sites prediction method is shown in Figure 1.

## 2 Materials and Methods

### 2.1 Datasets

In this study, the DNA-binding protein–drug complexes were derived from the sc-PDB database (Kellenberger et al., 2006). Until now, sc-PDB contains 16034 entries, which correspond to 4782 different proteins and 6326 different ligands. We obtained 17460 protein complexes from sc-PDB. After testing the consistency and deleting the redundancies, we obtained 120 DNA-binding protein–drug complexes, which include 107 drugs and 120 DNA-binding proteins.

A molecule is regarded as a ligand when it meets the following requirements: 1) it is not a water molecule, but is a small molecular weight molecule, such as drug, nucleotide, and endogenous ligand; 2) it has a limited solvent exposure to the surface. There is at least one residue atom less than 6.5 Å for any ligand atom; or 3) it does not covalently bind to peripheral proteins. Among them, the corresponding binding sites are formed by all DNA-binding protein residues with one or more atom within 6.5 Å of any drug atom. Therefore, 3853 binding sites were extracted from the protein–drug targets.

Among the total 120 DNA-binding protein–drug complexes containing 3,853 binding sites, a random sample (non-replacement) of 100 DNA-binding protein–drug complexes is chosen to train a model which contains 3,229 binding sites. These remaining 20 DNA-binding protein–drug complexes are used as independent test sets which contained 624 binding sites. The binding sites verified by experiments are represented as positive samples (i.e., binding sites), and all the remaining residues are labeled as negative samples (i.e., non-binding sites). To deal with the problem of class imbalance, sample scaling is the most direct method. We used random under-sampling method to select non-binding sites from all negative samples, and constructed a training set with a ratio of 1:1 for positive and negative samples to train the model.

### 2.2 Feature Extraction

#### 2.2.1 Position-Specific Scoring Matrices

There are some results showing that sequence-based calculation methods are of great use to predict binding sites (Wang et al., 2017; Wang et al., 2019c). The evolutionary information of the protein sequence is encoded by the position-specific scoring matrix (PSSM). The PSSM of each sequence uses PSI-BLAST (Altschul et al., 1997) to perform three iterations in the non-redundant protein sequence (nr) database, and the E-value is 0.001. PSSM is an L × 20 matrix, where the L row indicates the L amino acid residues contained by the protein sequence, and the 20 columns are the probabilities that each residue mutates to 20 local residues. The matrix is presented as follows:

$$PSSM=\left[\begin{array}{cc} P_{1,1} & \begin{array}{ccc} P_{1,2} & \cdots & P_{1,20} \end{array}\\ \begin{array}{c} P_{2,1}\\ \vdots \\ P_{L,1} \end{array} & \begin{array}{ccc} \begin{array}{c} P_{2,2}\\ \vdots \\ P_{L,2} \end{array} & \begin{array}{c} \cdots \\ \vdots \\ \ldots \end{array} & \begin{array}{c} P_{2,20}\\ \vdots \\ P_{L,20} \end{array} \end{array} \end{array}\right],$$ (1)

where the characteristics of each individual amino acid residue are described, and the PSSM feature has 20 dimensions.

#### 2.2.2 Residue Interaction Network Features

In the work of Wang et al. (Wang et al., 2019b; Wang et al., 2019a), we find it is closely related to the structural environment for the nucleic acid-binding protein. Instead of the nucleic acid sequences, local DNA and RNA structures are recognized by many proteins, such as G-quadruplexes, i-motifs, triplexes, left-handed DNA/RNA form, and many others. In addition, the studies of Bartas et al. have shown that protein structure depends on amino acid interactions (Bartas et al., 2021). Therefore, it is a great challenge to utilize these useful features to predict drug-binding sites, especially when the amino acids’s functional role is not fully recognized by the researchers currently. To deal with this conundrum, a residue interaction network is employed to depict protein structure in our work.

Residue interaction network (RIN) can represent the structure of a protein as a network, where amino acid residues are nodes, and the amino acids that interact with the amino acid are their edges. It is shown that RIN plays a useful role in bioinformatics’ applications (Pan et al., 2017; Astl and Verkhivker, 2019; Amitai et al., 2004; Li et al., 2011). Residues with high betweenness tend to have a lot of contacts (Sen et al., 2019). Moreover, betweenness is proved to be a better measure of the centrality in the interaction network, which can be interpreted as a correction to the number of contacts per residue. Residues with high closeness values interact directly or by a few intermediates with all other residues of the protein (Arumugam and Isacc, 2017). The nodes with high eigenvector centrality have a large influence on the overall information passing by flow, higher value, and better connectivity (Negre et al., 2018). Observing the previous studies, we find the functional importance of a protein site is closely related to its role in sustaining protein structure.

In this work, NAPS (Broto et al., 2019) is adopted to calculate the 7 topological features which mean the local features of the target residue include degree, closeness, betweenness, clustering coefficient, eccentricity, average nearest neighbor degree, and eigenvector centrality. The 7-dimensional network topology features are obtained through protein structure information.

We use betweenness(B) to indicate the ratio about all the shortest paths passing through a node and the total number of shortest paths. The formula can be described as

$$C_{b}\left(u\right)=\underset{s\neq u\in V}{\sum }\underset{t\neq u\in V}{\sum }\sigma_{st}\left(u\right)/\sigma_{st},$$ (2)

where $\sigma_{st}$ (*u*) is the number of shortest paths between *t* and *s* getting through the nodes *u*. $\sigma_{st}$ indicates the number of shortest paths between vertices *t* and *s*, and V indicates the set of all nodes.

Closeness (Cl) represents the centrality measure of the vertex, which is defined as the average geodesic distance from the node to all other vertices. The formula can be defined as

$$C_{cl}\left(u\right)=\left(n-1\right)/\underset{v\in V}{\sum }dist\left(u,v\right),$$ (3)

where dist (*u*, *v*) is the shortest path distance between nodes *v* and *u,* and *n* represents the number of nodes.

Eigenvector centrality (EC) is expressed as the component of the eigenvector corresponding to the largest eigenvalue of the adjacenct matrix. The formula is defined as follows:

$$xp=\frac{1}{\lambda }\overset{N}{\underset{q=1}{\sum }}A_{pq}x_{q},$$ (4)

where A_*pq*_ defines the strength of the physical correlation between nodes *p* and *q*, *λ* is the largest eigenvalue of A and xi is the eigenvector centrality of node p.

The eccentricity (E) signal that the shortest path distance of the node to the farthest node in the network. The formula can be expressed as follows:

$$C_{e}\left(u\right)=max(dist\left(u,v\right)).$$ (5)

Degree(D) is expressed as the number of edges incident to a vertex. This is calculated as

$$C_{d}\left(u\right)=\underset{v\in V}{\sum }A_{uv},$$ (6)

where A*uv* is the number of contacts between nodes *u* and *v*.

The clustering coefficient (CC) is a measure of the closeness of the neighbors of a vertex. It can be defined as

$$C_{cc}\left(u\right)=\lambda \left(u\right)/\gamma \left(u\right),$$ (7)

where λ(*u*) is the neighbors of *u* connected by an edge. The formula for γ(*u*) is

$$\lambda \left(u\right)=C_{d}\left(u\right)\left(C_{d}\left(u\right)-1\right)/2.$$ (8)

Average nearest neighbor degree (AN) is the average of the degree of its immediate neigh bours. It can be defined as

$$C_{an}\left(u\right)=\underset{v\in N\left(u\right)}{\sum }C_{d}\left(u\right)/N\left(u\right),$$ (9)

where N(*u*) is the neighbors of *u*.

### 2.3 Extreme Gradient Boosting Algorithm

The gradient boosting algorithm (Chen and Guestrin, 2016) retains the merits of the decision tree and constructs a set of strong learners from weak learners. The extreme gradient enhancement algorithm is an improvement of the gradient enhancement algorithm. Thus, the extreme gradient enhancement algorithm has a series of improvements in parallelism and prediction accuracy compared with the gradient enhancement algorithm.

In this study, we identify the binding sites and non-binding sites in DNA-binding protein–drug complexes. A two-category problem is proposed to identify binding sites and non-binding sites. We use feature vectors F*i* (F*i* = {f1, f2, ···, f*n*}, *i* = 1,2, ···, X) as the input and the class label y*i* (y*i* = {0,1}, *i* = 1,2, ···, X) as the output respectively, where X represents the number of rows of the feature vector, meanwhile 1 and 0 indicate binding sites and non-binding sites correspondingly. The XGBoost algorithm combines the techniques of classification and regression tree (CART) (Breiman et al., 1984) and a series of the gradient boosting machine.

### 2.4 Model Training

As mentioned before, we used three classification algorithms, i.e., XGBoost, SVM (Cherkassky, 1997), and CART to construct the proposed binding sites predictor in this study. For the purpose of training the classifier, we utilize the training data set to verify whether there is an improvement of the prediction accuracy. Then, we can get a better decision between binding sites and non-binding sites. Additionally, the models are trained with various feature combinations through different cross-validations. Among the three classifiers, XGBoost is considered as the best classifier, when the ratio of positive and negative samples of the training model is 1:1 and 10-fold cross-validation is performed.

### 2.5 Performance Evaluation

Classification performance is evaluated by accuracy (ACC), sensitivity (SEN), specificity (SPE), precision (PRE), and Matthews correlation coefficient (MCC). The area under the receiver operating characteristic curve (AUC) is used to evaluate the overall predictive quality of the binary model. The following formulas are used to determine ACC, SEN, SPE, PRE, and MCC, respectively:

$$ACC=\frac{T_{P}+T_{N}}{T_{P}+T_{N}+F_{P}+F_{N}},$$ (10)

$$SEN=\frac{T_{P}}{T_{P}+F_{N}},$$ (11)

$$SPE=\frac{T_{N}}{T_{N}+F_{N}},$$ (12)

$$PRE=\frac{T_{P}}{T_{P}+F_{P}},$$ (13)

$$MCC=\frac{T_{P}\times T_{N}-F_{P}\times F_{N}}{\sqrt{\left(T_{P}+F_{P}\right)\left(T_{P}+F_{N}\right)\left(T_{N}+F_{P}\right)\left(T_{N}+F_{N}\right)}}.$$ (14)

Among them, true positive (TP) represents the number of true protein–drug-binding sites that are predicted correctly; true negative (TN) represents the number of true non-binding sites that are correctly predicted; false negative (FN) represents the true protein–drug-binding sites and the number of points, these sites are designated as non-binding; false positive (FP) represents the number of true non-binding sites, these sites are designated as binding sites.

## 3 Result and Discussion

### 3.1 Performance Assessment of the Model

The 27-dimensional feature consists of two types, namely the residue interaction network (RIN) and position-specific scoring matrices (PSSMs) features. By means of the XGBoost algorithm, three different feature classifications are presented in our work. As shown in Table 1, we found that the network features appear better prediction performance between RIN and PSSM, with the highest ACC, MCC, and AUC values of 0.8057, 0.6607, and 0.8261, respectively. In addition, it can be seen from the table that the combined characteristics of PSSM and RIN achieve the best performance. Therefore, we can draw the conclusion that these two types of features may be complementary, and their combination can help predict the drug-binding sites and non-binding sites.

**TABLE 1:** Performance comparison of different feature combinations in XGBoost.

Feature group	ACC	PRE	SEN	SPE	MCC	AUC
PSSM	0.7262	0.8872	0.5348	0.9240	0.5139	0.7396
RIN	0.8057	0.9149	0.6607	0.9235	0.6563	0.8261
PSSM + RIN	0.8684	0.9246	0.8092	0.9304	0.7592	0.8990

In this study, the extreme gradient boosting classifier (XGBoost) is used to build the final model with 27 features. Through the experiment, we have found that the XGBoost can achieve the best performance comparing with SVM and CART. Based on 10-fold cross-validation on the training data set, the prediction results of XGBoost, SVM, and CART is shown in Table 2. The values of AUC obtained of XGBoost, SVM, and CART are 0.9464, 0.8141, and 0.8699, respectively. Compared with the SVM and CART methods, the XGBoost model is found to have higher ACC, SEN, MCC, and AUC scores, which improved the prediction performance.

**TABLE 2:** Performance of XGBoost in comparison with other classifiers on the combination feature set.

Method	ACC	PRE	SEN	SPE	MCC	AUC
SVM	0.7994	0.7735	0.8212	0.7962	0.6349	0.8141
CART	0.8386	0.8271	0.8492	0.8284	0.6936	0.8699
XGBoost	0.9316	0.9575	0.9110	0.9573	0.8844	0.9464
Independent testing	0.8894	0.9250	0.8950	0.9166	0.7538	0.7538

To evaluate the performance further, we compared XGBoost with SVM and CART, on the independent data sets (PSSM + RIN). XGBoost shows the best performance among the three classification methods for predicting the drug-binding sites. Therefore, we trained the training data set under the condition of jackknife cross-validation, 5-fold, and 10-fold cross-validation tests through the cross-validation test. From these three cross-validation tests, we selected the best classifier to optimize the performance of the three classification methods of SVM, CART, and XGBoost. We have found that the XGBoost exhibited the best performance than SVM and CART.

In order to test the performance of the model, we first applied the jackknife cross-validation test with the extreme gradient boosting classifier and achieved an AUC score 0.8593 with 83.21% accuracy for the training data set by using the combination feature (PSSM + RIN). From the results of 5-fold and 10-fold cross-validation tests, we observed that the performances are better than the jackknife cross-validation. In the 10-fold cross-validation test, the XGBoost classifier has an accuracy rate of 93.16% and the highest AUC value between SVM and CART. Table 3 shows the overall performance of XGBoost model in detail.

**TABLE 3:** Performance obtained from different cross-validation tests based on XGBoost algorithm.

CV-fold	ACC	PRE	SEN	SPE	MCC	AUC
Jackknife	0.8321	0.9104	0.7394	0.9206	0.6934	0.8593
5-fold	0.8583	0.9147	0.8003	0.9229	0.7404	0.8928
10-fold	0.9316	0.9575	0.9110	0.9573	0.8844	0.9464

### 3.2 Discussion of Network Topology Feature

From a biological point of view, the mutual constraint among residues is essential for the correct function of the appropriate structure (Balch et al., 2014). Seven well-established network topological features, eccentricity, closeness, clustering coefficient, betweenness, eigenvector centrality, degree, and average nearest neighbor degree are used to characterize DNA-binding proteins–drug sites in this work. Network topological features obtain the best performance. In order to determine the difference extent about DNA-binding proteins–drug sites in terms of such topological features, we perform an analysis. For the convenience of comparison, seven network topology features are normalized respectively, and we also analyze the difference between binding sites and non-binding sites in topological features.

**FIGURE 2:**
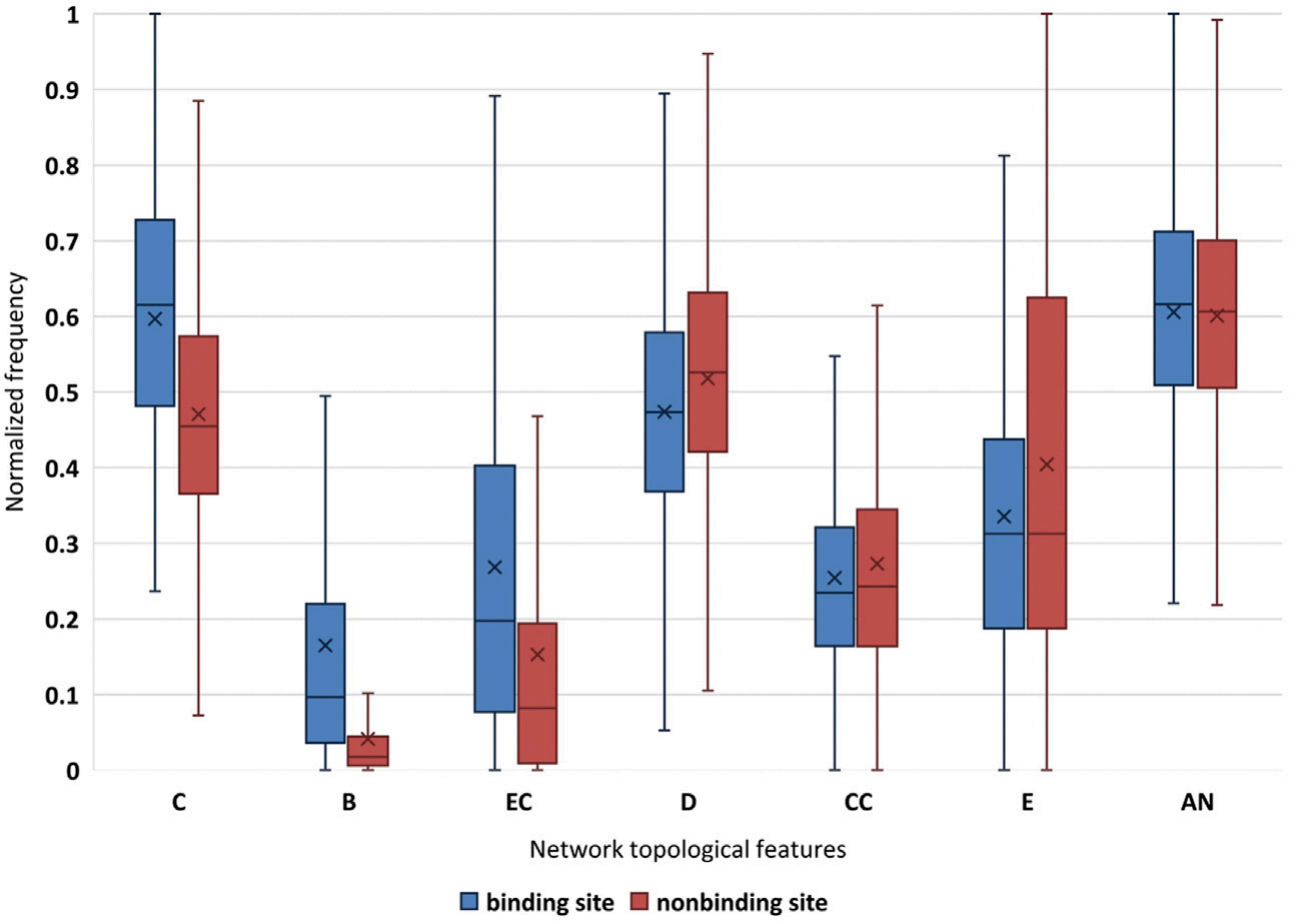
Normalization of network topological features for binding site and non-binding site. “×” is average value; C is closeness; B is betweenness; EC is eigenvector centrality; D is degree; CC is clustering coefficient; E is eccentricity; and AN is average nearest neighbor degree.

As shown in Figure 2, the closeness feature and betweenness feature of binding sites are significantly different from that of non-binding sites, followed by the feature of eigenvector centrality. From the basic aspects of protein structure, we understand a special local structure is often maintained by the cooperation of several residues. Figure 2 shows that DNA-binding protein–drug-binding sites may have more neighbors than non-binding sites. Obviously, the closeness of the binding sites is higher. Binding sites residues with high betweenness tend to have a high number of contacts. The high eigenvector centrality value of the binding site indicates that it has better contact with other residues in the network. And the mean value of binding sites is significantly higher than that of non-binding sites. For degree, eigenvector centrality, eccentricity, and the average nearest neighbor degree, the distributions of binding sites and non-binding sites are less distinct.

**FIGURE 3:**
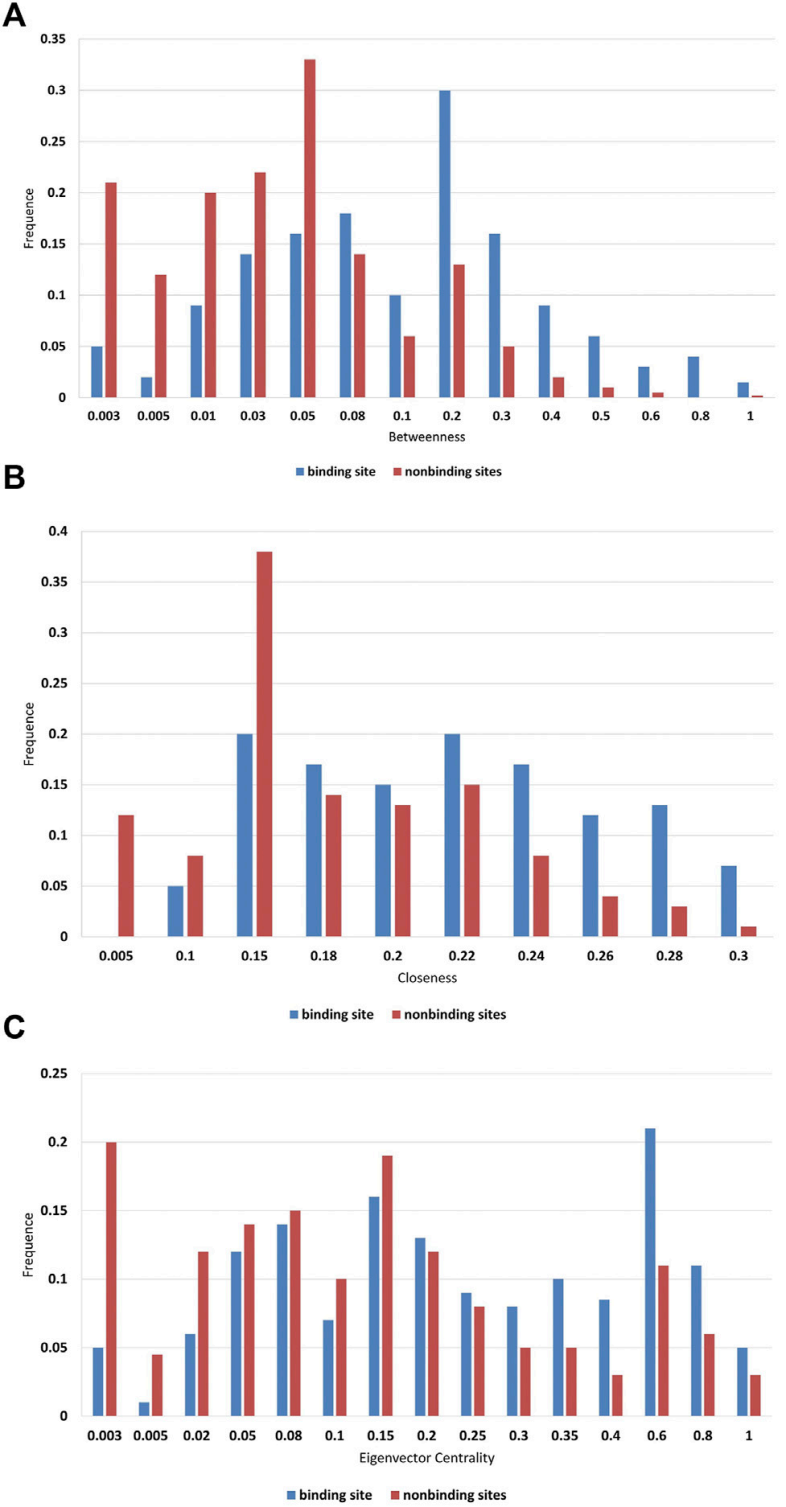
Frequency distributions of **(A)** betweenness; **(B)** closeness; and **(C)** eigenvector centrality.

Therefore, three well-established network topological features, closeness, betweenness, and eigenvector centrality are used to further characterize in Figure 3. We found that higher frequencies are detected for binding sites in the high scoring region obviously. In biology, key residues have a higher betweenness value, and this residue may interact with more residues (Figure 3A). According to these reports, closeness can indicate the functional role of residues. Thus, the fact that the high closeness value is observed at the binding site is not surprising (Figure 3B). In addition, the high eigenvector centrality value should focus on not only the nodes that are important per se, but the “neighborhood” of those nodes (Figure 3C). Therefore, it is reasonable to use these features to describe the structure and function of residues.

### 3.3 Analysis of Amino Acid Properties

**FIGURE 4:**
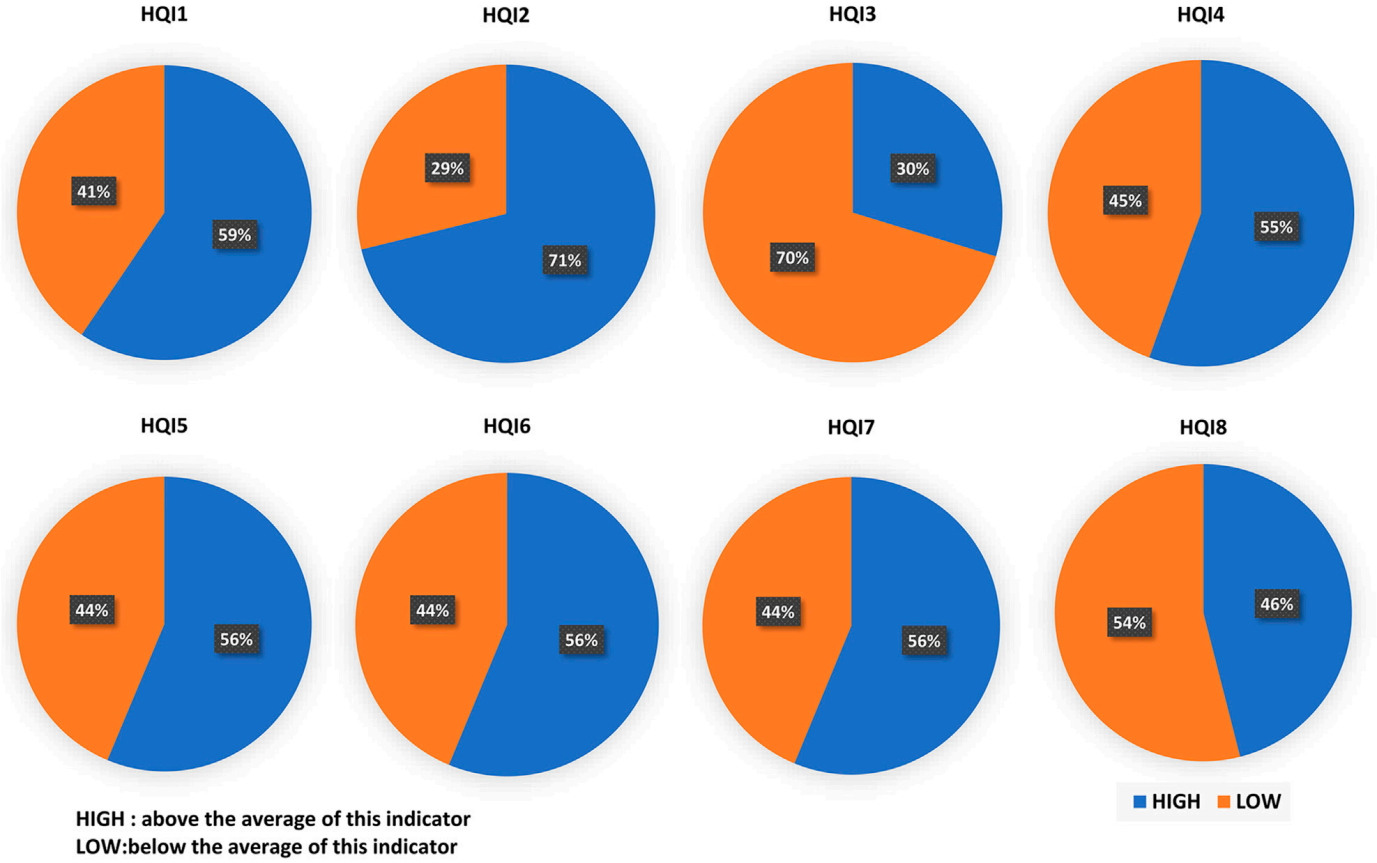
Proportion of eight high-quality amino acid indices (HQI) on the drug-binding sites. The eight indices from HQI1 to HQI8 denote electric charge properties, hydrophobicity, beta-strand propensities, alpha helix and turn propensities, volume, transmembrane residue propensities, amino acid composition, and relative partition energies, respectively. HIGH (blue color) denoted above the average of this indicator, while LOW (orange color) denoted below the average of this indicator.

Saha’s research divides the amino acid indexes in the AAindex database into 8 clusters, and 8 high-quality amino acid indexes are extracted from each cluster (Saha et al., 2012). We denoted the eight indices as HQI1 to HQI8, and analyzed the amino acid properties at the drug-binding site, which are displayed as Figure 4. In the electric charge property indices (HQI1), blue occupies 2/3 of the total. It is observed that drugs tend to act on positively charged amino acids (Porfireva et al., 2020). According to the electric field theory, dissimilar charges attract each other. This indicates that DNA-binding protein is more likely to bind to negatively charged drug molecules. It can be seen from HQI2 that hydrophobic amino acids account for 71%, which indicates that drugs tend to bind to hydrophobic amino acids (Lon and Martin, 2021). Usually, the surface of a protein is surrounded by hydrophilic amino acid residues, and the residues with hydrophobic side chains are located inside the molecule principally. This indicates that the binding process of DNA-binding protein and drug is more likely to occur inside the protein. HQI3 denotes beta-strand propensities, and HQI4 denotes alpha helix and turn propensities. The tendency of amino acids to form β-chain accounts for 30%, and the tendency to form α-helix accounts for 55%. In general, the amino acids in the complex that tend to form alpha helices and turns are more likely to interact with drugs. The proportion of large-volume amino acids (56%) is slightly more than that of the small-volume amino acids (44%) (Rani et al., 2016). Drugs are more likely to bind to larger amino acids, which indicates that sites with larger surface areas are more likely to interact with drugs. HQI6 represents transmembrane residue propensities. Amino acid in the complexes is favored to be localized in the transmembrane regions. The region of the protein sequence that spans the cell membrane is usually an α-helical structure, which corresponds to the conclusion of HQI4. HQI7 represents the amino acid compositions of intracellular proteins (Abe and Nitta, 1984). This means that the residues at the binding site (such as Leu, Phe, Ala, and Val) are easier to interact with the drug. The higher ratio the relative partition energies (HQI8) of the residues ranks, the easier the amino acid contacts with other residues. This conclusion shows that the residues at the binding site contacting more other residues can bind the drug better.

### 3.4 Analysis of Drug–Ligand

**FIGURE 5:**
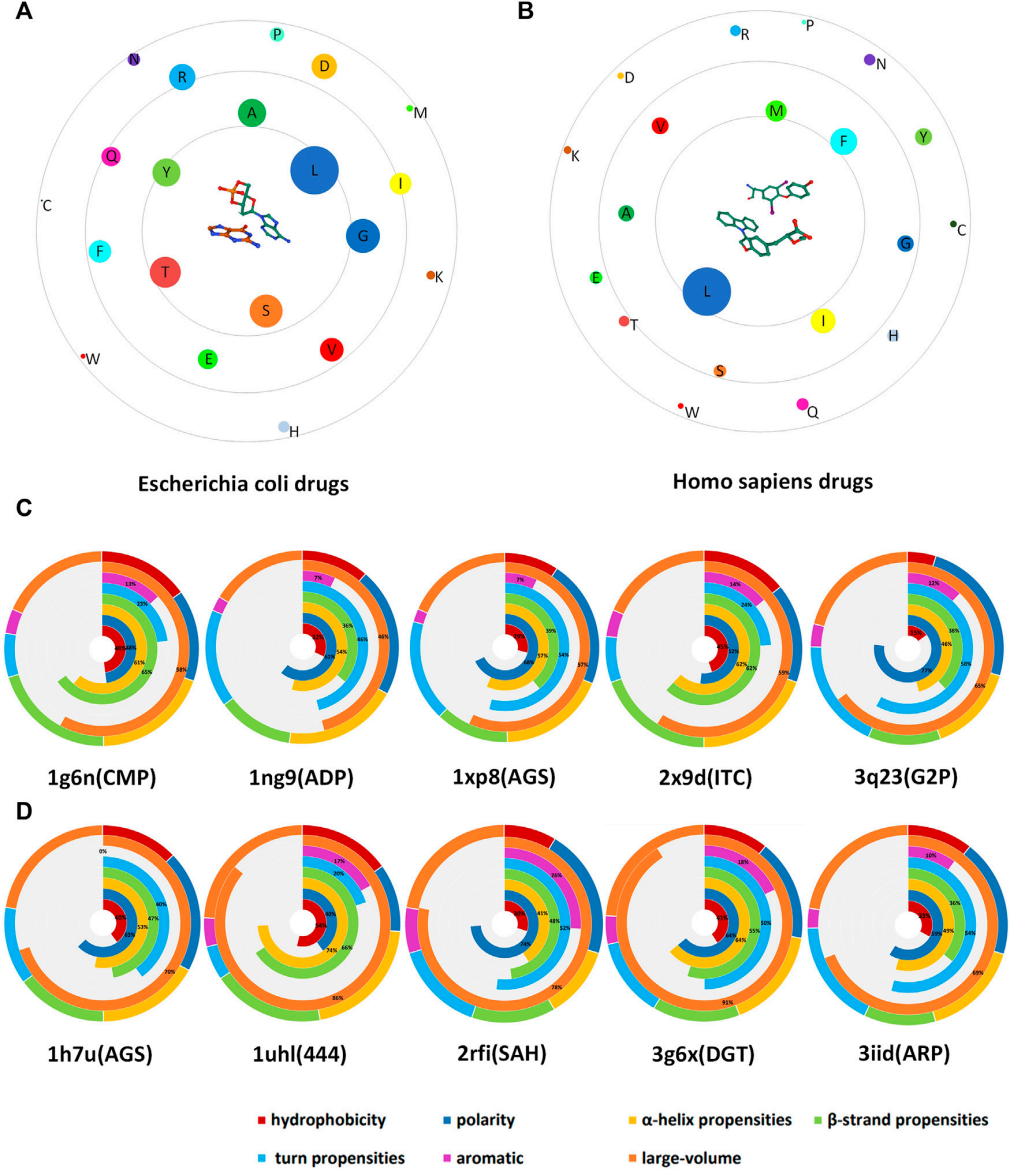
Schematic diagram of drug-binding amino acid preferences. **(A)** is the drug ligand of *Escherichia coli*; **(B)** is the drug ligand of homo sapiens. In the figure, the drug is located in the center, and the binding site residues are distributed around, and we used circles of different sizes and colors to indicate the number and type of amino acids. **(C)** is the case of *Escherichia coli* drug-associated protein; **(D)** is the case of Homo sapiens drug-associated protein. Different colors are used to indicate the proportion of the characteristics.

In this study, we obtained a total of 3,853 drug-binding sites. In order to explore the propensity of different drug-binding sites to bind to amino acid residues, we divided the drug ligands into 19 categories according to the biological types of DNA-binding protein–drug complexes. We selected two types of organisms that have the largest proportion: Homo sapiens and *Escherichia coli*. Among them, Homo sapiens contains 1,546 amino acid sites, and *Escherichia coli* contains 692 amino acid sites. We presented the relationship diagram of drug ligands’ tendency to bind to amino acids and select some cases as shown in Figure 5. From Figures 5A,B it can be seen that the drug (choose one of the drugs as the representative) is located in the center, the 20 amino acids are represented by circles with different colors. The size of the circle indicates the binding ability of the amino acid to the drug. At the same time, the distance between the amino acid and the drug indicates the tendency of binding to the drug. The Figure 5A is drugs of *Escherichia coli*, in general, the drugs tend to bind Leu, Gly, and Ser, and combine amino acid property analysis. We found that amino acids that tend to form a helix and favor to be localized in the transmembrane regions are more likely to bind to drugs. Through the analysis of the Figure 5B (drug of Homo sapiens), we found that the drugs of homo sapiens are easier to bind to Leu, Ile, Phe, and Met. These amino acids carry more electric charge, are less hydrophobic, prefer to form alpha helices and turns, and have a larger volume.

In order to verify the correctness of our conclusions, 5 drugs belonging to the *Escherichia coli* biotype (Figure 5C) and 5 drugs belonging to the Homo sapiens biotype (Figure 5D) were selected, and radial graphs were drawn respectively. The outer circle represents the proportion of a certain property of the type of drug ligands that tend to bind to amino acids in all properties. The seven inner circles are represented by different colors, respectively indicating the hydrophobicity, polarity, alpha helix propensities, beta-strand propensities, turn propensities, aromatic amino acids, and the proportion of large-volume amino acids. At the bottom of the figure, the first four digits are the ID name of the PDB and the parentheses are the name of the drug ligand. In addition to the conclusions drawn from Figures 5A,B, we found that these two classes of drugs have a common feature of low binding ability to aromatic amino acids.

## 4 Conclusion and Prospect

Predicting the drug-binding sites accurately plays an essential role to understand the underlying molecular recognition mechanism in DNA-binding protein complexes. In this research, we extracted the drug-binding sites from DNA-binding protein–drug complexes. We utilized sequence information to obtain PSSM and used network information to obtain RIN to predict the binding site of drug ligands. Then, we used the XGBoost method to construct the prediction model. The experiment results show that our method performed better than the other methods on both training set and independent set. In this work, in order to study the correlation among residues, we provided a network to represent the protein structure. In addition, network topological features appropriately reflect the role of DNA-binding protein–drug-binding sites in not only local structures, but also global ones by exploiting their correlation with other residues. Through the analysis of the physicochemical properties of the drug-binding site, we found that residue-binding sites carry more positive electric charge, are more hydrophobic, prefer to form alpha helices and turns, and large amino acid volumes are easier to bind drug ligands. In the future, we expect there is a protein structure network with finer residue interactions that can reflect the structure and function of the residue in the protein more accurately. It is also believed that with the identification of more DNA-binding proteins–drug-binding sites , the volume of the training set will be expanded. As technologies continue to mature in machine learning, there will be more excellent binding site prediction methods.

## Data Availability

The original contributions presented in the study are included in the article/Supplementary Material, further inquiries can be directed to the corresponding authors.
